# Supplementary material for: Spatial Analysis of Shared Risk Factors between Pleural and Ovarian Cancer Mortality in Lombardy (Italy)
Source: Int J Environ Res Public Health. 2022 Mar 15;19(6):3467. doi: 10.3390/ijerph19063467 (PMC8949464; doi:10.3390/ijerph19063467)
Supplement: Supplementary file 1 [file ijerph-19-03467-s001.zip › ijerph-1599733-supplementary.pdf]

Supplementary material

Table S1: Mean of the posterior distribution of the heterogeneity and clustering hyperparameters and the heterogeneity/clustering ratio

disease	Clustering SD	Heterogeneity SD	Odds heterogeneity:clustering
MM	0.6026	0.09541	1:6.4
Ovarian Cancer	0.08587	0.06199	1:1.4

Table S2: WAIC and median calibrated Kullback–Leibler divergence (Calibrated KL) for all the fitted models.

Model	Description	UK	VK	U	V	WAIC	Calibrated KL
M7	Uk U Vk V	X	X	X	X	5190.261	0.500
M5	Uk Vk V	X	X		X	5192.567	0.526
M2	Uk U Vk	X	X	X		5202.911	0.698
M4	U Vk V		X	X	X	5210.906	0.561
M3	Uk U V	X		X	X	5219.626	0.578
M9	Uk V	X			X	5223.190	0.698
M1	Uk Vk	X	X			5235.503	0.698
M6	U V			X	X	5258.971	0.730
M8	U Vk		X	X		5269.865	0.796

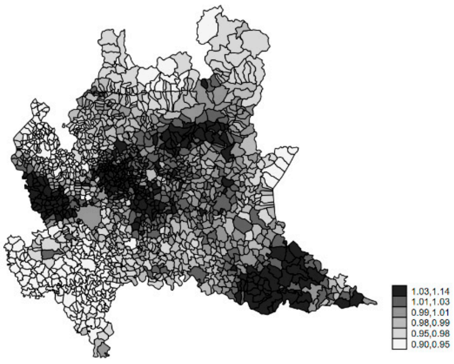

Figure S1. Spatial choropleth map of Bayesian smoothed standardized mortality ratios (SMR) for ovarian cancer. Lombardy Region, 2000-2018. Relative scale (sextiles).

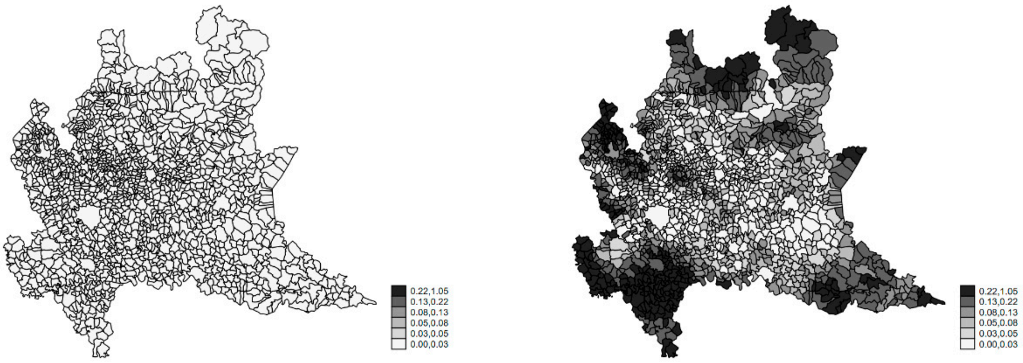

Figure S2: Maps of calibrated KL by municipalities for model 4 for pleural cancer (left panel) and ovarian cancer (right panel).

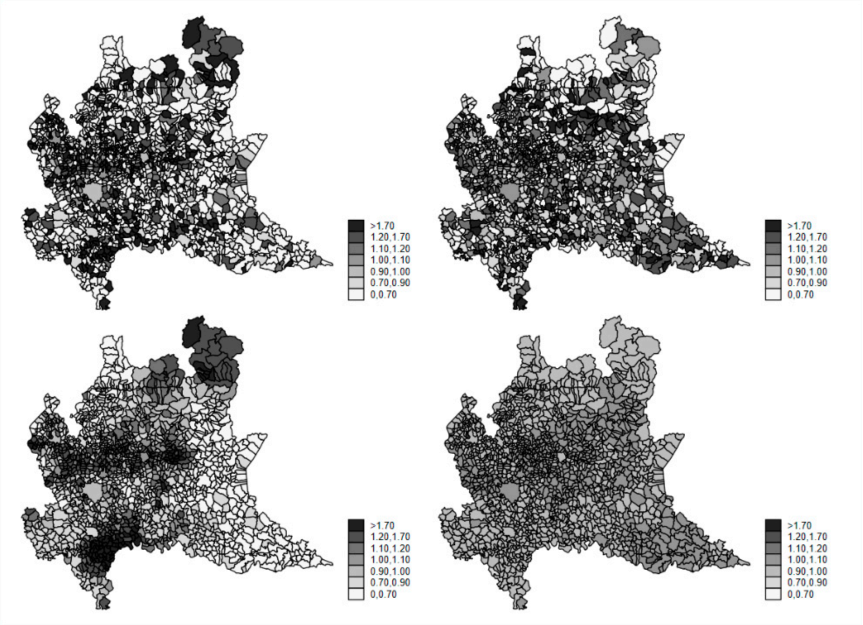

Figure S3. Spatial choropleth map of raw (upper panels) and Bayesian smoothed (lower panels) standardized mortality rates of malignant mesothelioma (left panels) and ovarian cancer (right panels). Lombardy Region, 2000-2018. Absolute scale.

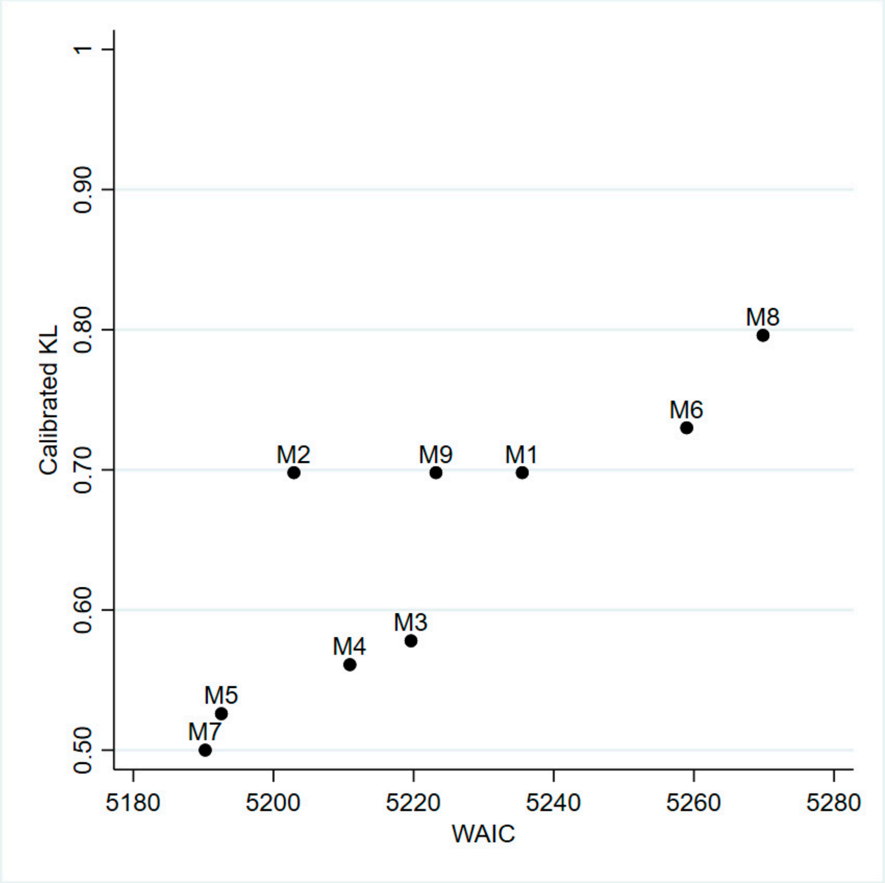

Figures S4 Scatter plot of Calibrated Kullback-Leibler divergence (KL) vs Predictive Accuracy measured by the widely applicable or Watanabe-Akaike Information Criterion (WAIC) for each fitted Bayesian model. Calibrated KL is calculated with regard to model 7 – the more complex and best-fitting model. Ovarian cancer and MM by the municipality. Lombardy Region, 2000-2018.

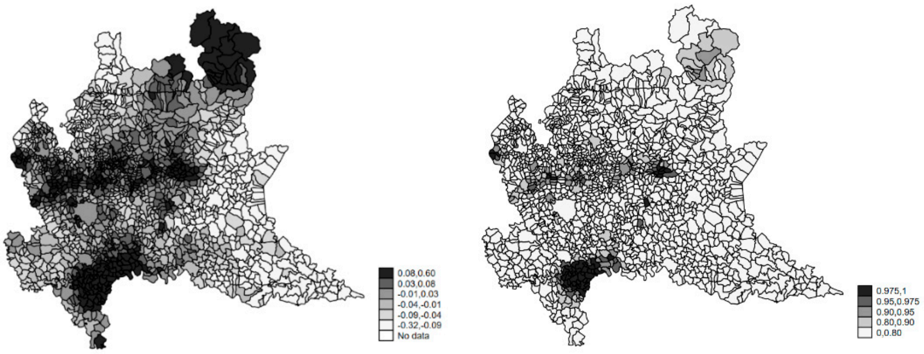

Figure S5. Maps of shared clustering terms (U) between MM and ovarian cancer from model 4 (M4) (left panel) and Posterior Probability of Direction of Effect - Prob(U>0) (right panel). Lombardy Region, 2000-2018.
